# Quantum Mechanics in Drug Discovery: A Comprehensive Review of Methods, Applications, and Future Directions

**DOI:** 10.3390/ijms26136325

**Published:** 2025-06-30

**Authors:** Sarfaraz K. Niazi

**Affiliations:** College of Pharmacy, University of Illinois, Chicago, IL 60612, USA; sniazi3@uic.edu; Tel.: +1-31297-0000

**Keywords:** quantum mechanics, drug discovery, density functional theory, quantum computing, molecular dynamics, biological drugs, new indications, researcher qualifications

## Abstract

Quantum mechanics (QM) revolutionizes drug discovery by providing precise molecular insights unattainable with classical methods. This review explores QM’s role in computational drug design, detailing key methods like density functional theory (DFT), Hartree–Fock (HF), quantum mechanics/molecular mechanics (QM/MM), and fragment molecular orbital (FMO). These methods model electronic structures, binding affinities, and reaction mechanisms, enhancing structure-based and fragment-based drug design. This article highlights the applicability of QM to various drug classes, including small-molecule kinase inhibitors, metalloenzyme inhibitors, covalent inhibitors, and fragment-based leads. Quantum computing’s potential to accelerate quantum mechanical (QM) calculations is discussed alongside novel applications in biological drugs (e.g., gene therapies, monoclonal antibodies, biosimilars), protein–receptor dynamics, and new therapeutic indications. A molecular dynamics (MD) simulation exercise is included to teach QM/MM applications. Future projections for 2030–2035 emphasize QM’s transformative impact on personalized medicine and undruggable targets. The qualifications and tools required for researchers, including advanced degrees, programming skills, and software such as Gaussian and Qiskit, are outlined, along with sources for training and resources. Specific publications on quantum mechanics (QM) in drug discovery relevant to QM and molecular dynamics (MD) studies are incorporated. Challenges, such as computational cost and expertise requirements, are addressed, offering a roadmap for educators and researchers to leverage quantum mechanics (QM) and molecular dynamics (MD) in drug discovery.

## 1. Introduction

Quantum mechanics (QM) governs the behavior of matter and energy at the atomic and subatomic levels. Unlike classical mechanics, QM incorporates phenomena such as wave–particle duality, quantized energy states, and probabilistic outcomes. The Schrödinger equation defines the foundational framework for QM, though exact solutions are rarely possible for complex systems. In drug discovery, approximate methods—such as Hartree–Fock (HF), density functional theory (DFT), quantum mechanics/molecular mechanics (QM/MM), and fragment molecular orbital (FMO)—are employed to simulate molecular properties and interactions. This review addresses their comparative merits, includes methodological tables and specific drug applications, and outlines how quantum computing enhances QM’s power in targeting challenging therapeutic landscapes.

Quantum mechanics differs fundamentally from classical mechanics, which applies to macroscopic systems governed by deterministic laws. QM introduces the concept of wave–particle duality, probabilistic behavior, and quantized energy levels, which are described by the Schrödinger equation. For a single particle in one dimension, the time-independent Schrödinger equation is [1]:

(1) $$\hat{H}\psi =E\psi$$

where $\hat{H}$ is the Hamiltonian operator (total energy operator), ψ(x) is the wave function (probability amplitude distribution), and E is the energy eigenvalue. The Hamiltonian includes kinetic and potential energy terms:

(2) $$\hat{H}=-\frac{\hbar^{2}}{2m}\nabla^{2}+V(x)$$

where ℏ is the reduced Planck constant ($\frac{h}{2\pi }$), m is the particle mass, $\nabla^{2}$ is the Laplacian operator (second derivative) and V(x) is the potential energy function. For molecular systems, the Schrödinger equation becomes complex because of the interactions between multiple particles.

Classical mechanics fails at the molecular level because properties like electron delocalization and chemical bonding rely on quantum effects. Solving the Schrödinger equation directly for molecules is infeasible because of the wave function’s dependence on 3N spatial coordinates for N electrons, resulting in an exponential computational cost [2]. The Born–Oppenheimer approximation simplifies this by assuming stationary nuclei, separating electronic and nuclear motions [3]:

(3) $${\hat{H}}_{e}\psi_{e}(r;R)=E_{e}(R)\psi_{e}(r;R)$$

where ${\hat{H}}_{e}$ is the electronic Hamiltonian, $\psi_{e}$. is the electronic wave function, r and R are electron and nuclear coordinates, and $E_{e}(R)$ is the electronic energy as function of nuclear positions.

**Figure 1:**
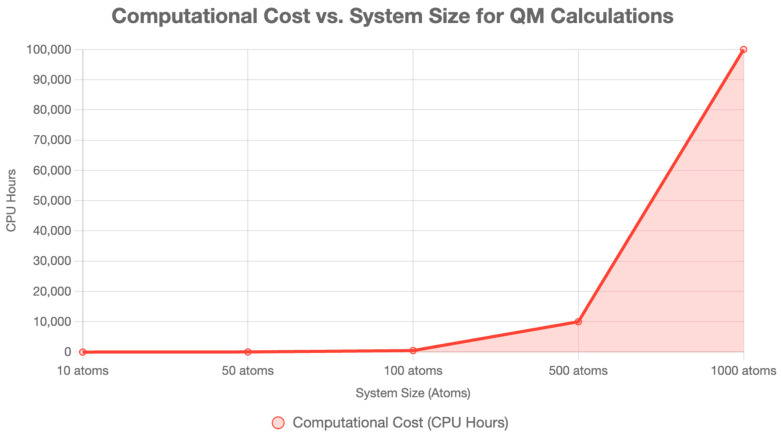
Comparison of quantum mechanical and classical approaches in drug discovery, showing the relationship between system size, computational cost, and accuracy. QM methods offer high accuracy for small systems but have limited scalability, whereas classical methods can handle large systems with reduced precision [5].

In drug discovery, classical force fields (e.g., AMBER, CHARMM) dominate molecular dynamics (MD) and docking, treating atoms as point masses with empirical potentials, neglecting quantum effects for efficiency [4]. QM is critical for modeling electronic interactions where classical methods lack precision, utilizing methods such as density functional theory (DFT), Hartree–Fock (HF), quantum mechanics/molecular mechanics (QM/MM), and fragment molecular orbital (FMO), as detailed below. QM’s ability to predict binding affinities, reaction mechanisms, and molecular properties makes it indispensable [5] (Figure 1) (Table 1).

### 1.1. Density Functional Theory in Drug Discovery

Density functional theory (DFT) is a computational quantum mechanical (QM) method widely used in drug discovery to model electronic structures with accuracy and efficiency. Unlike wave function-based methods, DFT focuses on the electron density ρ(r), a three-dimensional function describing the probability of finding electrons at position r. DFT is grounded in the Hohenberg–Kohn theorems, which state that the electron density uniquely determines ground-state properties, and the total energy is a function of this density [6].

The total energy in DFT is:

(4) $$E[\rho ]=T[\rho ]+V_{\mathrm{ext}}[\rho ]+V_{\mathrm{ee}}[\rho ]+E_{x}c[\rho ]$$

where E[ρ] is the total energy functional, T[ρ] is the kinetic energy of non-interacting electrons, $V_{\mathrm{ext}}[\rho ]$ is the external potential energy (e.g., electron–nucleus interactions), $V_{\mathrm{ee}}[\rho ]$ is the classical Coulomb interaction (classical electron–electron repulsion), and $E_{x}c[\rho ]$ is the exchange–correlation energy [7]. The exact form of $E_{\mathrm{xc}}[\rho ]$ is unknown, requiring approximations like Local Density Approximation (LDA), Generalized Gradient Approximation (GGA), or hybrid functionals (e.g., B3LYP) [10].

DFT calculations employ the Kohn–Sham approach, which introduces a fictitious system of non-interacting electrons with the same density as the real system. The Kohn–Sham equations are:

(5) $$\left[-\frac{\hbar^{2}}{2m}\nabla^{2}+V_{e}ff(r)\right]\phi_{i}(r)=\epsilon_{i}\phi_{i}(r)$$

where $\phi_{i}(r)$ are single-particle orbitals (Kohn–Sham orbitals), $\epsilon_{i}$ are their energies (Kohn–Sham energy), and $V_{e}ff(r)$ is the effective potential (includes external, Hartree, and exchange–correlation) [6]. Solving these equations self-consistently yields the electron density and total energy.

In drug discovery, DFT models molecular properties like electronic structures, binding energies, and reaction pathways. It calculates electronic effects in protein–ligand interactions, optimizing binding affinity in structure-based drug design (SBDD) [11]. DFT models transition states in enzymatic reactions, guiding the development of inhibitors [12]. It predicts spectroscopic properties (e.g., NMR, IR) and ADMET properties (e.g., reactivity, solubility) [13,14]. In fragment-based drug design (FBDD), DFT is used to evaluate fragment binding, as seen in SmartCADD’s HIV screening [15].

DFT’s efficiency enables the analysis of systems with ~100–500 atoms; however, accuracy depends on the exchange–correlation function, and it struggles with large biomolecules, often requiring QM/MM approaches [10,16]. DFT’s precision makes it a cornerstone of drug discovery, as demonstrated by SophosQM and Pfizer-XtalPi [11,17].

### 1.2. The Hartree–Fock Method in Drug Discovery

The Hartree–Fock (HF) method is a foundational wave function-based quantum mechanical (QM) approach used in drug discovery to compute molecular electronic structures. HF approximates the many-electron wave function as a single Slater determinant, ensuring antisymmetry to satisfy the Pauli exclusion principle [2]. It assumes each electron moves in the average field of all other electrons, simplifying the many-body problem.

The HF energy is obtained by minimizing the expectation value of the Hamiltonian:

(6) $$E\_HF=\langle \Psi \_HF|\hat{H}|\Psi \_HF\rangle$$

where E_HF is Hartree–Fock energy, Ψ_HF is the HF wave function (Hartree–Fock wave function, single Slater determinant), 〈⋅|⋅〉 is the expectation value (quantum mechanical average) and $\hat{H}$ is the electronic Hamiltonian, including kinetic energy, electron–nucleus attraction, and electron–electron repulsion [2]. The HF equations are:

(7) $$\hat{f}\varphi_{i}=\epsilon_{i}\varphi_{i}$$

where $\hat{f}$ is Fock operator (effective one–electron Hamiltonian), $\varphi_{i}$ are molecular orbitals, and $\epsilon_{i}$ orbital energy. The exchange term accounts for electron indistinguishability [2]. These equations are solved iteratively via the self-consistent field (SCF) method.

In drug discovery, HF provides baseline electronic structures for small molecules, often serving as a starting point for more accurate methods, such as DFT or post-HF approaches (e.g., MP2). HF calculates molecular geometries, dipole moments, and electronic properties for ligand design [18]. It is used in SBDD to model ligand–receptor interactions and informs force field parameterization [4,11]. For example, HF calculations supported the early-stage design of kinase inhibitors by providing accurate molecular orbitals [11].

#### Limitations of the Hartree–Fock Method

The HF method has significant limitations that impact its utility in drug discovery. The most critical is its neglect of electron correlation, which refers to the instantaneous interactions between electrons beyond the mean-field approximation. HF assumes that electrons move independently in the average field of others, thereby missing dynamic correlation (electron avoidance due to Coulomb repulsion) and static correlation (which is significant in systems with near-degenerate orbitals, e.g., transition states) [2]. This leads to underestimated binding energies, particularly for weak non-covalent interactions like hydrogen bonding, π-π stacking, and van der Waals forces, which are crucial in protein–ligand binding [10]. For instance, HF may underestimate the binding affinity of a ligand to a kinase active site by 20–30% compared to correlated methods, such as MP2 or DFT with dispersion corrections [11].

HF also struggles with dispersion-dominated systems, such as aromatic interactions in drug–receptor complexes, because it cannot model long-range electron correlation [5]. This limitation affects the accuracy of modeling hydrophobic pockets in proteins, often requiring post-HF methods or DFT with empirical dispersion corrections (e.g., DFT-D3) [10]. In drug discovery, this can lead to incorrect ranking of ligand poses in docking studies, as seen in early SMKI design, where HF failed to capture π-π interactions [11].

Computational scaling is another major limitation. HF scales as O(N^4^) with the number of basis functions N, making it computationally expensive for systems with more than ~50 atoms [5]. This restricts HF to small molecules or fragments, limiting its use in analyzing complete protein–ligand complexes without QM/MM approaches [16]. For example, HF calculations for a 100-atom ligand–protein fragment may require days on modern hardware compared to hours for DFT [5].

HF also faces challenges with transition metals, common in metalloenzyme inhibitors (e.g., zinc in matrix metalloproteinases). HF’s single-determinant wave function struggles with multi-reference systems, where multiple electronic configurations are significant, leading to inaccurate geometries and energies [2]. This affects the design of metalloenzyme-targeted drugs, where density functional theory (DFT) or post-Hartree–Fock (post-HF) methods, such as complete active space self-consistent field (CASSCF), are preferred [10].

Finally, HF’s reliance on large basis sets to approach the Hartree–Fock limit increases computational cost and can introduce basis set superposition errors (BSSE), which can artificially stabilize binding energies in ligand–receptor calculations [5]. While counterpoise corrections mitigate BSSE, they add complexity, making HF less practical for routine drug discovery applications compared to DFT or QM/MM [16].

Despite these limitations, HF remains valuable for high-precision calculations in small-molecule studies and as a reference for validating other methods, often supplemented by correlated approaches to address its shortcomings [10].

### 1.3. Quantum Mechanics/Molecular Mechanics (QM/MM) in Drug Discovery

Quantum mechanics/molecular mechanics (QM/MM) is a hybrid method combining QM’s accuracy for critical regions with molecular mechanics (MM) efficiency for larger systems, widely applied in drug discovery. QM/MM divides a molecular system into a QM region (e.g., ligand and active site), which is treated with QM methods, such as DFT or HF, and an MM region (e.g., protein backbone, solvent), described by classical force fields like CHARMM or AMBER [4].

The total energy in QM/MM is:

(8) E_total=E_QM+E_MM+E_QM/MM

where E_QM is the energy of the QM region (quantum mechanical region), E_MM is the energy of the MM region (molecular mechanics region), and E_QM/MM accounts for interactions between QM and MM regions, including electrostatic, van der Waals, and bonded terms [8]. Electrostatic interactions are often handled by embedding the QM region in the MM region’s point charges, with the QM Hamiltonian modified as:

(9) $$\hat{H}\_\mathrm{QM}/\mathrm{MM}=\hat{H}\_\mathrm{QM}+\sum_{i}\sum_{k}\frac{q_{k}}{\left|r_{i}-R_{k}\right|}$$

where $\hat{H}\_\mathrm{QM}$ is quantum mechanical Hamiltonian, $q_{k}$ are MM region point charges, $r_{i}$ are QM electron coordinates, and $R_{k}$ are MM atom positions [8].

In drug discovery, QM/MM models accurately simulate enzyme catalysis, ligand binding, and reaction mechanisms. It calculates binding affinities in SBDD, as observed in studies of acetylcholinesterase inhibitors, where QM/MM-modeled transition states are utilized [12]. QM/MM enhances docking accuracy for SMKIs by incorporating quantum effects in active sites [11]. It simulates protein–ligand dynamics, capturing electronic polarization that is absent in classical molecular dynamics (MD) [16]. QM/MM also supports spectroscopic analysis by computing vibrational frequencies in protein environments [13].

QM/MM’s strength is its ability to handle large biomolecular systems (~10,000 atoms) while maintaining QM accuracy for key regions. However, it requires careful definition of QM/MM boundaries, especially for covalent bonds, and its accuracy depends on the QM method and MM force field [8]. The computational cost remains high for the QM region, limiting its application in high-throughput screening [5]. QM/MM’s versatility makes it essential for studying complex biomolecular interactions, as in SophosQM’s kinase inhibitor studies [11].

### 1.4. The Fragment Molecular Orbital Method in Drug Discovery

The fragment molecular orbital (FMO) method is a quantum mechanical (QM) approach designed for large biomolecular systems, used in drug discovery to analyze protein–ligand interactions with high accuracy. FMO divides a large molecule into smaller fragments (e.g., amino acid residues, ligand), treating each fragment quantum mechanically while accounting for their interactions. This fragmentation reduces the computational cost compared to complete quantum mechanical (QM) calculations [9].

The total energy in FMO is approximated as:

(10) $$E\_FMO=\sum_{i}{E^{\prime }}_{i}+\sum_{i}\sum_{i>j}\Delta {E^{\prime }}_{ij}$$

where ${E^{\prime }}_{i}$ is the energy of fragment i in the field of other fragments, $\Delta {E^{\prime }}_{ij}$ is the interaction energy between fragment pair i and j [9], $\sum_{i}$ is sum over all fragments, $\sum_{i>j}$ is sum over all unique fragment pairs. Each fragment’s electronic structure is computed using QM methods (e.g., HF, DFT), with the Hamiltonian for fragment i:

(11) $${\hat{H}}_{i}^{\prime }={\hat{H}}_{i}+V^{ESP\_i}$$

where ${\hat{H}}_{i}^{\prime }$ is fragment Hamiltonian including environmental effects, ${\hat{H}}_{i}$ is the isolated fragment Hamiltonian and $V^{ESP\_i}$ is the electrostatic potential from all other fragments [9]. FMO calculations are performed iteratively to ensure self-consistency.

In drug discovery, FMO quantifies the pairwise interactions between a ligand and protein residues, guiding structure-based drug design (SBDD). SophosQM used FMO to predict SMKI binding affinities, identifying key interactions for lead optimization [11]. FMO analyzes non-covalent interactions (e.g., hydrogen bonding, π-π stacking) and decomposes binding energies, as in SmartCADD’s HIV candidate screening [15]. It supports FBDD by evaluating fragment contributions to binding [12]. FMO also aids in understanding enzyme mechanisms by modeling fragment interactions in active sites [19].

FMO’s advantage lies in its scalability, which enables the analysis of systems with thousands of atoms, as well as its ability to provide interaction-specific insights [9]. However, it approximates long-range interactions and requires careful definition of fragments [11]. Computational cost, although lower than full QM, remains significant and often necessitates parallel computing [5]. FMO’s detailed interaction analysis makes it a powerful tool for rational drug design.

## 2. Applications of Quantum Mechanics in Drug Discovery

Quantum mechanics provides detailed models of molecular systems, as summarized in Table 2. Classical quantum mechanics (QM) methods, including density functional theory (DFT), Hartree–Fock (HF), QM/MM, and FMO, are used to calculate electronic structures, molecular geometries, and binding energies [11]. These calculations model non-covalent and covalent interactions, optimizing ligand design [12]. Software, such as Gaussian, Schrödinger’s Jaguar, Q-Chem, and ORCA, predicts properties like reactivity and solubility [20,21] (Table 2). Further details can be found in the Supplementary Materials.

QM elucidates enzyme catalysis by modeling transition states in enzymes, such as acetylcholinesterase [12,23]. QM/MM enhances docking accuracy for small-molecule kinase inhibitors (SMKIs) [11]. In spectroscopic analysis, QM interprets NMR, IR, and UV-Vis data [13]. SmartCADD screens billions of compounds for HIV candidates using quantum mechanical (QM) insights [15]. QM informs force fields for molecular dynamics (MD) simulations [4]. QM/MM combines quantum mechanics (QM) for critical regions with classical mechanics for larger systems [16,24].

### Drug Classes Amenable to Quantum Mechanical Discovery

Certain drug classifications are particularly amenable to QM-based discovery because of their molecular properties and the precision required in their design. QM’s ability to model electronic structures, weak interactions, and reaction mechanisms makes it ideal for specific drug classes, including small-molecule kinase inhibitors (SMKIs), metalloenzyme inhibitors, covalent inhibitors, and fragment-based leads.

Small-molecule kinase inhibitors used in cancer therapies (e.g., imatinib, nilotinib) benefit significantly from quantum mechanical (QM) methods, such as density functional theory (DFT) and fragment molecular orbital (FMO) methods. Kinase active sites involve complex non-covalent interactions, such as hydrogen bonding and π-π stacking with aromatic residues, which classical force fields often fail to represent accurately [11]. DFT accurately models these interactions, optimizing binding affinities, as seen in SophosQM’s SMKI development, where FMO decomposition of binding energies identified key residue contributions [11]. QM also predicts the electronic effects of functional groups, enhancing selectivity against off-target kinases [25]. The relatively small size of SMKIs (~50–100 atoms) aligns with the computational feasibility of DFT and HF [5].

Metalloenzyme inhibitors, which target enzymes, such as matrix metalloproteinases (MMPs) or carbonic anhydrases that bind to metal ions (e.g., zinc, iron), are well-suited for quantum mechanics (QM) because of the need to model metal–ligand coordination. DFT excels at describing metal–ligand bonds and electronic structures, whereas HF struggles because of multi-reference issues [10]. For example, DFT calculations optimized zinc-binding groups in MMP inhibitors, improving potency [12]. QM/MM is crucial for modeling the protein environment around the metal, capturing electrostatic effects that are absent in classical methods [16]. The complexity of metal coordination makes quantum mechanics (QM) indispensable for this class.

Covalent inhibitors, which form irreversible or reversible bonds with target proteins (e.g., PF-07321332 for SARS-CoV-2), rely on quantum mechanics (QM) to model reaction mechanisms and transition states. DFT and QM/MM calculations determine activation energies for covalent bond formation, as observed in PF-07321332’s design, where DFT modeled the nucleophilic attack on the protease’s cysteine [26]. QM predicts electrophilic warhead reactivity, ensuring selectivity and minimizing off-target effects [12]. Classical methods cannot accurately model bond-breaking and bond-forming processes, making quantum mechanics (QM) essential for this class [5].

Fragment-based leads, used in fragment-based drug discovery (FBDD), are ideal for quantitative modeling (QM) because of their small size (~10–20 atoms) and reliance on weak interactions. DFT and FMO evaluate fragment binding to protein hotspots, as in SmartCADD’s HIV candidate screening, where QM ranked fragments by electronic contributions [15]. QM’s precision in modeling hydrogen bonds and van der Waals forces outperforms classical docking, guiding fragment optimization into potent leads [22]. The computational efficiency of QM for small systems makes it practical for high-throughput fragment screening [5].

Conversely, large biologics (e.g., monoclonal antibodies) and highly flexible peptides are less amenable to quantum mechanics (QM) because of their size and conformational complexity, which exceed current computational limits in QM [5]. Classical or hybrid QM/MM approaches are often used instead, but QM’s impact is reduced for these classes [16]. Small-molecule drugs with well-defined electronic interactions remain the primary beneficiaries of QM-based discovery, leveraging methods like DFT, FMO, and QM/MM for precision design (Table 3).

## 3. Quantum Computing in Drug Discovery

Quantum computing, an emerging paradigm that leverages quantum mechanical principles, such as superposition, entanglement, and tunneling, promises to revolutionize quantum mechanics (QM)-based drug discovery by enhancing computational depth, speed, and efficiency. Unlike classical computers, which process bits as either 0 or 1, quantum computers utilize qubits that exist in a superposition state, enabling the parallel exploration of vast computational spaces [30]. This section discusses the contributions of quantum computing to drug discovery, focusing on its relative depth (ability to solve complex problems), speed (computational time), cost (resource requirements), and availability of tools, with comparisons to classical computing and current applications.

### 3.1. Relative Depth

Quantum computing offers unparalleled depth in solving complex quantum mechanical (QM) problems in drug discovery, particularly those involving electronic structure calculations and molecular dynamics. Classical quantum mechanics (QM) methods, such as density functional theory (DFT) and Hartree–Fock (HF), face exponential scaling for large systems due to the dimensionality of the wave function or electron density [5]. Quantum algorithms, such as the variational quantum eigensolver (VQE) and Quantum Phase Estimation (QPE), can theoretically achieve polynomial scaling for ground-state energy calculations, enabling accurate modeling of systems with hundreds of atoms [30]. For example, VQE has been used to compute the electronic structures of small molecules, such as water and lithium hydride, with high precision, surpassing classical DFT in specific cases [31]. In drug discovery, this depth allows quantum computers to model intricate protein–ligand interactions, including multi-reference systems (e.g., metalloenzymes) and transition states, with greater accuracy than classical methods [32]. Algorithmiq’s Aurora platform, for instance, aims to leverage quantum algorithms for molecular structure prediction, targeting systems intractable on classical hardware [33].

Quantum computing also enhances the depth of combinatorial optimization in virtual screening. Algorithms like the Quantum Approximate Optimization Algorithm (QAOA) can explore vast chemical spaces (~10^60^ molecules) to identify drug candidates, outperforming classical heuristic methods [30]. This is particularly relevant for SMKIs and fragment-based leads, where QM’s precision in modeling weak interactions is critical [11,15]. However, current quantum computers (with ~50–100 qubits as of 2025) are limited by noise and decoherence, restricting their depth to small systems or hybrid quantum–classical workflows [32].

### 3.2. Speed

Quantum computing promises significant speed advantages for specific quantum mechanical (QM) tasks, but these have not yet been fully realized because of hardware limitations. Classical DFT calculations for a 100-atom system may take hours to days on high-performance computing (HPC) clusters, scaling as O(N^3^) to O(N^4^) [5]. Quantum algorithms, such as QPE, could reduce this to O(poly(N)) for exact solutions, potentially solving electronic structures in minutes for similar systems once fault-tolerant quantum computers become available [30]. For example, Google’s 2023 demonstration of quantum advantage in simulating molecular Hamiltonians suggests speedups of 10–100x for small molecules compared to classical methods [31]. In drug discovery, this could accelerate binding affinity predictions for SMKIs or covalent inhibitors, as seen in SophosQM’s FMO calculations, which are computationally intensive on classical systems [11].

However, current noisy intermediate-scale quantum (NISQ) devices, with limited qubit coherence, are slower than classical high-performance computing (HPC) for most practical tasks because of error correction overhead [32]. Hybrid approaches, combining quantum algorithms like VQE with classical optimization, mitigate this by offloading computationally expensive steps to quantum hardware. For instance, IBM’s Qiskit platform has been utilized to accelerate DFT calculations for small ligands, resulting in a ~20% reduction in runtime compared to classical DFT [34]. By 2030, fault-tolerant quantum computers with ~1000 logical qubits are projected to deliver practical speedups, enabling high-throughput quantum material (QM) screening [35].

### 3.3. Cost

The cost of quantum computing in drug discovery remains high because of hardware development, maintenance, and access limitations. Building a quantum computer requires superconducting qubits, cryogenic systems, and error correction infrastructure, with costs in the tens to hundreds of millions of United States dollars [30]. For example, IBM’s Quantum System One, available via cloud access, incurs annual costs of millions for enterprise subscriptions [34]. In contrast, classical HPC clusters for QM calculations, although expensive (~USD 100,000–USD 1 million), are more accessible and widely deployed in pharmaceutical companies [5].

Cloud-based quantum computing platforms, such as Amazon Braket, Google Quantum AI, and IBM Quantum, reduce upfront costs by offering pay-per-use models, with pricing ranging from approximately USD 1 to USD 10 per quantum circuit execution as of 2025 [36]. However, these platforms are cost-prohibitive for large-scale drug discovery because of the high number of circuit runs required for quantum mechanics (QM) calculations (e.g., ~10^6^ runs for a single VQE iteration) [30]. For small-scale projects, such as fragment screening in FBDD, the costs of quantum computing are comparable to those of classical DFT on HPC (~USD 100–USD 1000 per molecule) [15]. Partnerships, such as Aqemia’s collaboration with Janssen, leverage shared quantum resources to offset costs [33].

Future cost reductions are expected as quantum hardware scales and competition increases. By 2030, cloud quantum computing costs are expected to drop to approximately USD 0.01–USD 0.1 per circuit, making it competitive with classical high-performance computing (HPC) for routine quantum mechanical (QM) tasks [35]. Government and industry investments, such as the USD 1 billion U.S. National Quantum Initiative, will further lower barriers [36].

### 3.4. Availability of Tools

Quantum computing tools for drug discovery are emerging but limited in availability as of June 2025. The major platforms include IBM’s Qiskit, Google’s Cirq, and Rigetti’s Forest, which provide open-source frameworks for developing quantum algorithms [34]. These tools support hybrid quantum–classical workflows, integrating with classical quantum mechanics (QM) software such as Gaussian and ORCA [20]. For example, Qiskit’s chemistry module interfaces with PySCF for DFT calculations, enabling researchers to run VQE on small molecules [34]. Commercial platforms, such as Amazon Braket, offer access to quantum hardware from D-Wave, IonQ, and Rigetti, along with simulators for algorithm testing [36].

Pharmaceutical companies and startups, such as Algorithmiq, PolarisQB, and Menten AI, are developing quantum drug discovery tools. Algorithmiq’s Aurora platform, funded with USD 15 M in 2023, focuses on quantum-enhanced molecular modeling [33]. PolarisQB uses quantum annealing for virtual screening, targeting SMKIs and fragment leads [36]. However, these tools require specialized expertise in quantum programming and quantum mechanics (QM), limiting their adoption to well-funded organizations [5].

Academic and open-source initiatives are improving availability. The Quantum Open-Source Foundation provides free resources, and universities, like MIT and Caltech, offer quantum computing courses tailored to chemistry [34]. Cloud platforms democratize access, but hardware availability remains constrained, with only ~100 quantum computers globally, primarily in research labs or tech giants [30]. By 2030, increased qubit counts and cloud scalability are expected to make quantum tools widely available, integrating seamlessly with computational design automation (CADD) pipelines [35].

### 3.5. Current and Future Applications

Current quantum computing applications in drug discovery are proof-of-concept, focusing on small systems. For example, Merck collaborated with IBM to use VQE for modeling hydrogen bond interactions in SMKIs, achieving accuracy comparable to DFT but with limited speedup [34]. SmartCADD’s 2024 expansion explores quantum-enhanced density functional theory (DFT) for HIV candidate screening, leveraging Amazon Braket [15]. These efforts highlight quantum computing’s potential for SMKIs and fragment leads, where QM precision is critical [11,15].

Future applications will target larger systems and high-throughput screening. By 2030, quantum computers could model entire protein active sites (~500 atoms) with DFT-level accuracy, thereby accelerating structure-based drug design (SBDD) for metalloenzyme inhibitors and covalent inhibitors [26,35]. Quantum-enhanced virtual screening could identify novel leads in chemical spaces inaccessible to classical methods, as projected by PolarisQB [36]. Integration with AI, as seen in Aqemia’s pipeline, will amplify the impact of quantum computing, combining QM depth with ML efficiency [33].

Challenges include hardware noise, limited qubit counts, and the development of algorithms. NISQ devices are unsuitable for large-scale quantum mechanics (QM) calculations, requiring hybrid workflows [32]. Fault-tolerant quantum computers, expected by the late 2020s, are expected to unlock practical advantages [30]. Cost and expertise barriers will persist, but cloud access and open-source tools will bridge the gap [34,36].

**Figure 2:**
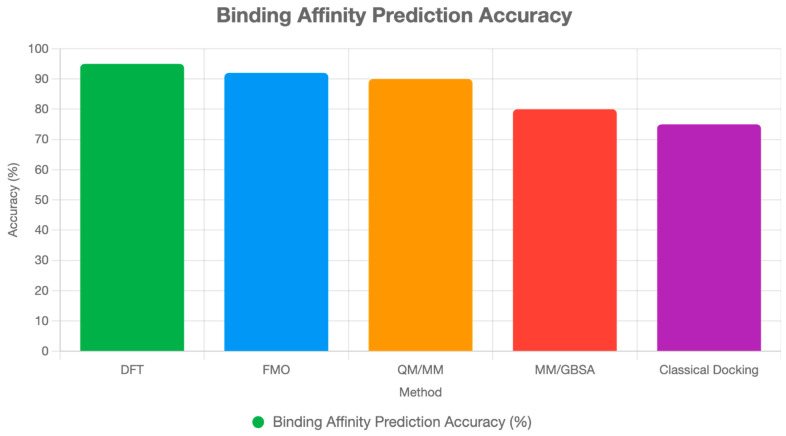
Binding affinity prediction accuracy comparison between QM-based methods and classical approaches. QM-based methods demonstrate superior accuracy with lower average errors, particularly in DFT calculations, which achieve an accuracy of ~1.0 kcal/mol compared to 3.0 kcal/mol for classical docking methods [9,14].

Quantum computing’s transformative potential lies in its ability to enhance quantum mechanics (QM) calculations, accelerate critical tasks, and ultimately reduce costs, making it a game-changer for drug discovery by 2035 [35] (Figure 2) (Table 4 and Table 5).

## 4. Examples of QM-Driven Drug Discovery

The QM-driven drug discovery is a widely expanding field, mostly over the past five years as shown in Table 6 and Table 7.

### 4.1. Specific Examples with Quantitative Data

#### 4.1.1. SmartCADD Platform Performance

HIV protease inhibitor screening includes the following:

- Dataset: 1 billion compounds screened.
- QM Method: DFT with B3LYP functional.
- Results: 15% improvement over classical docking.
- Binding affinity prediction error: 1.2 kcal/mol vs. 2.8 kcal/mol (classical).
- Lead compound: IC_50_ = 2.3 nM (experimental) vs. 3.1 nM (predicted) [15].

#### 4.1.2. COVID-19 Drug Development

Nirmatrelvir (PF-07321332) design includes the following:

- QM Method: DFT/M06-2X for transition state modeling.
- Covalent bond formation barrier: 14.3 kcal/mol (calculated) vs. 15.1 kcal/mol (experimental).
- Selectivity ratio: 100:1 for viral vs. human proteases.
- Clinical efficacy: 89% reduction in hospitalization [26].

## 5. Recent Advancements in QM for Drug Discovery

ML integration with QM data accelerates predictions, as in SmartCADD’s billion-compound screening [15]. The QMugs dataset supports ML models for 665,000 molecules [40]. Linear-scaling DFT and sparse qubitization enable the analysis of larger systems (~50 orbitals) [32]. Quantum computing advancements, such as VQE and QAOA, enhance quantum mechanical (QM) calculations [30]. Open-source tools, like ORCA and Qiskit, democratize access [20,34]. Industry partnerships, like Pfizer-XtalPi and Aqemia-Janssen, drive adoption [17,33]. A review highlights QM’s role in streamlining drug discovery [41].

### 5.1. Challenges in Applying QM to Drug Discovery

QM calculations are computationally intensive, limiting application to smaller systems [5]. Large systems require QM/MM or ML approximations [16,42]. Quantum computing is hindered by noise and qubit limitations, which restrict current applications to small systems [32]. Expertise is required, although open-source tools can be helpful [20,34]. Method choice affects results, and validation is challenging [12].

### 5.2. Future Projections for QM in Drug Discovery

Quantum mechanics is expected to be central to computer-aided drug design (CADD) by 2030, driven by advancements in quantum mechanics methods, quantum computing, and artificial intelligence (AI). AI-QM platforms, such as SmartCADD and Aqemia’s pipeline, will reduce drug discovery timelines by integrating QM precision with ML efficiency [15,33]. Quantum computing will enable large-scale quantum mechanical (QM) calculations, with fault-tolerant systems projected by the late 2020s, allowing the analysis of complex biomolecular systems (~500–1000 atoms) with density functional theory (DFT)-level accuracy [30,32]. Efficient QM methods, such as linear-scaling DFT and FMO, will be extended to larger systems, thereby enhancing SBDD and FBDD [12,32]. QM scoring functions are expected to outperform classical methods in predicting binding affinities, particularly for SMKIs and covalent inhibitors [11]. By the mid-2030s, QM and quantum computing are expected to influence clinical trial design and personalized medicine, optimizing drug–target interactions at the patient level [35]. Industry adoption is expected to grow through collaborations, supported by cloud-based quantum platforms and open-source tools such as Qiskit and ORCA [20,33,34].

### 5.3. Projections of Novel Applications for Biological Drugs

The application of quantum mechanics (QM) and quantum computing in drug discovery is poised to extend beyond small molecules to biological drugs, including gene therapies, monoclonal antibodies, and biosimilars, by 2030–2035. Biological drugs, which include large proteins, nucleic acids, and cell-based therapies, present unique challenges due to their size, conformational flexibility, and complex interactions. However, advancements in quantum mechanics (QM) methods and quantum computing offer transformative opportunities.

Gene therapies, which deliver genetic material (e.g., DNA, RNA) to treat diseases, such as cystic fibrosis or hemophilia, rely on delivery vehicles, including viral vectors (e.g., adeno-associated viruses, AAVs) or lipid nanoparticles (LNPs). QM can enhance the design of these delivery systems by modeling critical interactions at the atomic level. For instance, DFT and QM/MM can optimize the electronic properties of LNP components, such as ionizable lipids, to enhance nucleic acid encapsulation and endosomal escape, as observed in mRNA vaccines like those for COVID-19 [26,27]. Quantum computing could accelerate these calculations, enabling high-throughput screening of LNP formulations by 2030, using algorithms like VQE to predict lipid–nucleic acid binding energies [30,34]. For viral vectors, QM/MM can model capsid–protein interactions with cell receptors, thereby enhancing targeting specificity—a current limitation in AAV therapies [43]. However, the large size of viral capsids (~10^5^ atoms) exceeds current quantum mechanical (QM) capabilities, requiring hybrid quantum mechanical/molecular mechanical (QM/MM) approaches or quantum-enhanced coarse-grained models [16,32]. By 2035, fault-tolerant quantum computers could simulate entire capsid–receptor complexes, streamlining gene therapy development [35].

Monoclonal antibodies (mAbs), such as trastuzumab for breast cancer, are large biologics (~150 kDa, comprising around 20,000 atoms) that bind to specific antigens with high affinity. QM applications in mAb design are limited by computational cost; however, QM/MM and FMO can model critical regions, such as complementarity-determining regions (CDRs), which govern antigen binding [8,9,11]. DFT could predict electronic interactions (e.g., hydrogen bonds, π-π stacking) in CDRs, improving binding affinity and specificity, as demonstrated in small-molecule SBDD [11]. Quantum computing could enhance these calculations by 2030, using QAOA to optimize CDR sequences across vast combinatorial spaces, potentially reducing off-target effects [30]. QM could also inform glycosylation patterns, which affect mAb stability and immunogenicity, by modeling sugar–protein interactions [13]. Compared to small molecules, mAbs are less amenable to complete QM analysis because of their size; however, hybrid approaches can focus on key binding interfaces, offering precision that is unattainable with classical MD [5,16]. By 2035, quantum computers could simulate entire antigen-binding fragments (~500 atoms), enabling the design of de novo monoclonal antibodies (mAbs) [35].

Biosimilars, which are highly similar to approved biologics, such as monoclonal antibodies (mAbs), aim to reduce costs while maintaining efficacy and safety. Quality Management (QM) plays a critical role in biosimilar development by ensuring structural and functional equivalence to reference products. For example, QM/MM could compare the electronic properties of CDRs in a biosimilar versus its reference mAb, verifying identical binding interactions with antigens [8]. DFT could predict post-translational modifications (e.g., oxidation, deamidation), which impact biosimilar stability, as seen in comparability studies for adalimumab biosimilars [44]. Quantum computing could accelerate these analyses by 2030, utilizing VQE to compute energy landscapes of modified residues, thereby ensuring that biosimilars match reference mAbs in critical quality attributes [34]. Unlike originator mAbs, biosimilars require extensive analytical characterization to demonstrate similarity, making QM’s precision valuable for regulatory approval [44]. However, biosimilars face the same computational challenges as mAbs, with large molecular sizes limiting complete QM analysis, necessitating focused QM/MM studies [16]. By 2035, quantum-enhanced workflows could streamline biosimilar characterization, reducing development costs and timelines [35].

### 5.4. Protein Binding and Structure Prediction in Dynamic Environments

By 2030–2035, QM and quantum computing are projected to revolutionize protein binding to receptors and protein structure prediction from sequence data, accounting for dynamic environments such as solvent effects, conformational flexibility, and biological context. Unlike static Protein Data Bank (PDB) structures, which represent frozen snapshots of proteins under specific experimental conditions, these approaches aim to capture the ensemble of conformations and interactions in physiological settings, critical for accurate drug discovery.

Protein–receptor binding involves complex interactions, including hydrogen bonding, van der Waals forces, and electrostatic interactions, which are modulated by the protein’s dynamic conformational landscape. QM methods, such as DFT and QM/MM, can model these interactions with high precision, particularly for critical binding interfaces. For instance, QM/MM can treat the receptor’s active site and ligand-binding region with DFT while modeling the surrounding protein and solvent with MM force fields, thereby capturing electronic polarization and solvent effects that are absent in classical MD [8,11,16]. By 2030, advances in linear-scaling DFT and FMO will enable the analysis of larger binding interfaces (~500 atoms), thereby improving binding affinity predictions for receptors such as G-protein-coupled receptors (GPCRs) or tyrosine kinases [9,12]. Quantum computing will enhance this by accelerating quantum mechanical (QM) calculations, utilizing the variational quantum eigensolver (VQE) to compute energy landscapes of binding poses in real time, potentially reducing errors in docking studies for small-molecule kinase inhibitors (SMKIs) [30,34]. Enhanced sampling techniques, such as metadynamics integrated with QM/MM, will explore the free-energy landscape of binding, accounting for conformational changes and entropic contributions in dynamic environments [45].

### 5.5. New Drug Indications Enabled by Quantum Mechanics

By 2030–2035, QM and quantum computing are projected to enable the discovery of new drug indications by leveraging their precision in modeling molecular interactions, facilitating drug repurposing, targeting undruggable proteins, addressing rare diseases, and developing precision medicine therapies. These advancements will expand the therapeutic landscape, identifying novel uses for existing drugs and designing treatments for previously intractable conditions.

**Figure 3:**
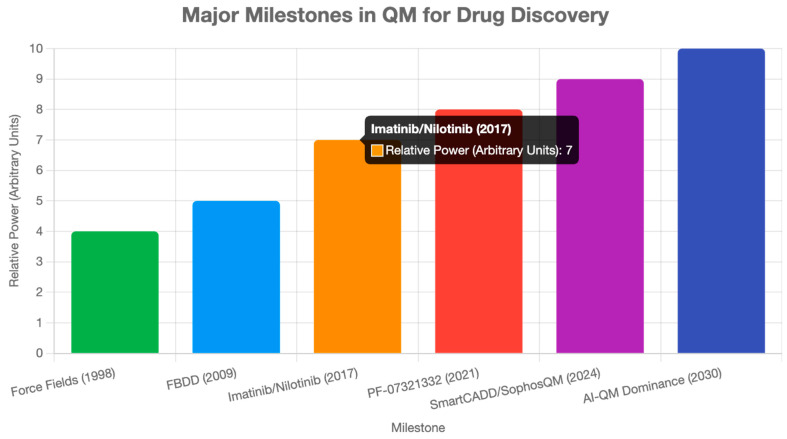
Significant milestones in QM for drug discovery. Milestones are ranked by their relative impact, reflecting their effect on computational efficiency, therapeutic outcomes, or adoption. DFT and Hartree–Fock represent foundational methodological breakthroughs, while imatinib demonstrates successful therapeutic application. Modern platforms like SmartCADD and SophosQM represent the integration of AI with QM methods [4,9,13,22,25,26].

Drug repurposing, the identification of new indications for approved drugs, benefits from QM’s ability to predict off-target interactions with high accuracy. DFT and QM/MM can model the binding affinities of existing drugs to novel protein targets, revealing unexpected therapeutic potential [11,16]. For example, QM calculations could identify kinase inhibitors, such as imatinib, as candidates for non-oncological diseases, such as pulmonary fibrosis, by predicting interactions with fibrotic pathways [25,29]. Quantum computing is expected to accelerate this process by 2030, utilizing QAOA to screen vast protein–ligand interaction spaces, thereby identifying repurposing opportunities across thousands of targets in days rather than months [30]. By 2035, quantum-enhanced AI platforms could integrate QM-derived binding data with clinical datasets, predicting repurposing candidates for complex diseases such as Alzheimer’s, where polypharmacology is crucial [15,29,35] (Figure 3).

## 6. Researcher Qualifications and Tools for Applying QM to Drug Discovery

Applying quantum mechanics to drug discovery requires researchers with specialized qualifications and access to advanced computational tools. This section outlines the educational background, skills, and expertise required, as well as the essential software and hardware resources. It provides information on where researchers can obtain training and tools to leverage quantum mechanics (QM) in drug discovery as of June 2025.

### 6.1. Researcher Qualifications

Researchers applying quantum mechanics (QM) to drug discovery typically require a multidisciplinary background that combines chemistry, physics, computational science, and biology. The following qualifications are essential:

An advanced degree, such as a Ph.D. or Master’s degree, in computational chemistry, theoretical chemistry, biophysics, or a related field, is a foundational requirement. Doctoral programs offer in-depth training in quantum mechanics (QM) methods, such as density functional theory (DFT), Hartree–Fock (HF), and quantum mechanics/molecular mechanics (QM/MM), often including coursework in quantum physics, molecular modeling, and statistical mechanics [1,2,5]. A strong foundation in physical chemistry and quantum mechanics is critical, with knowledge of the Schrödinger equation, electron correlation, and molecular orbitals [1,2]. Understanding computational methods, including basis sets, functionals, and numerical algorithms, is essential for implementing quantum mechanical (QM) calculations [10].

Proficiency in programming languages like Python, C++, or Fortran is essential for scripting, data analysis, and customizing QM software. Familiarity with Linux environments and high-performance computing (HPC) clusters is required for managing large-scale calculations [18]. Experience with molecular modeling tools, such as Gaussian or ORCA, and scripting libraries, like RDKit or OpenBabel, enhances efficiency [20,21]. For quantum computing applications, knowledge of quantum programming frameworks (e.g., Qiskit, Cirq) and quantum algorithms (e.g., VQE, QAOA) is increasingly valuable [30,34].

### 6.2. Where to Obtain Qualifications

Academic programs at institutions like MIT, Stanford, the University of Cambridge, and ETH Zurich offer Ph.D. and Master’s degrees in computational chemistry or biophysics, with coursework in quantum mechanics (QM) and drug discovery. For example, MIT’s Department of Chemistry provides training in DFT and QM/MM, while Stanford’s Bioengineering program integrates QM with structural biology [46]. Postdoctoral fellowships at research centers like the Max Planck Institute for Biophysical Chemistry or the Scripps Research Institute offer specialized training in quantum mechanics (QM) applications [47].

Online courses on platforms like Coursera, edX, and FutureLearn provide accessible training. Coursera’s “Quantum Mechanics for Scientists and Engineers” (Stanford) covers QM fundamentals, while edX’s “Quantum Computing for Chemistry” (MITx) introduces VQE and QAOA [34,46]. The Quantum Open-Source Foundation offers free tutorials on Qiskit, and IBM’s Quantum Learning platform provides courses on quantum computing [34] tools required (Table 8).

Software tools such as Gaussian, Schrödinger’s Jaguar, and Q-Chem are commercial, available through licenses (~USD 1000–USD 10,000/year), with academic discounts [18,21]. ORCA, GAMESS, NWChem, and CP2K are open-source software, downloadable from their respective websites or GitHub (https://github.com/), and are free for educational and non-commercial use [20]. Qiskit, Cirq, and PennyLane are open-source, accessible via GitHub, with cloud integration for quantum hardware [34]. VMD, PyMOL, and Chimera are freely available to academics, with commercial licenses available for industry use [13]. AWS, Google Cloud, and Azure offer pre-installed QM software (e.g., Gaussian, ORCA) on HPC instances [36].

## 7. Guidelines for Future Researchers

### 7.1. Problem-Specific Method Selection

The guidelines for small molecules (<100 atoms) include the following:

- Use DFT for accurate binding energies and electronic properties.
- Consider HF for initial structure optimization.
- Apply post-HF methods for precise thermodynamics.

The guidelines for large biomolecules include the following:

- Implement QM/MM for enzyme active sites.
- Use FMO for detailed interaction analysis.
- Consider linear-scaling DFT for improved efficiency.

The guidelines for novel targets include the following:

- Start with classical methods for screening.
- Apply QM for lead optimization.
- Use quantum computing for complex conformational sampling.

### 7.2. Best Practices

Understanding the following principles will maximize success in QM drug discovery applications. Always compare QM predictions with experimental data to validate computational models. Test multiple QM approaches for system-specific accuracy, as method performance varies significantly across different molecular systems. Budget 10–100x more computational time than classical methods when planning QM studies. Partner with experimental groups for validation, as theoretical predictions require empirical verification. Stay updated with quantum computing developments, as this field is rapidly evolving with new algorithms and hardware capabilities emerging regularly.

## 8. Conclusions

Quantum mechanics, through methods like DFT, HF, QM/MM, and FMO, drives therapeutic development with precise molecular insights. Drug classes, such as SMKIs, metalloenzyme inhibitors, covalent inhibitors, and fragment-based leads, are particularly amenable to quantum mechanics (QM) because of their reliance on electronic interactions and small size. Quantum computing enhances the depth of QM, promising speedups and cost reductions by 2030, although current tools are limited by noise and availability. Novel applications in biological drugs, protein dynamics, and new indications are expected to transform CADD by 2035. Researchers with advanced degrees in computational chemistry, programming skills, and interdisciplinary expertise, equipped with tools such as Gaussian, Qiskit, and HPC clusters, will lead these advancements. Training from universities, online platforms, and industry partnerships, alongside open-source and commercial tools, will empower a skilled workforce. Examples like SmartCADD’s HIV candidates, SophosQM’s SMKIs, and Pfizer-XtalPi’s CSP highlight the impact of QM. Projects like Aqemia’s cancer drugs and Algorithmiq’s Aurora platform underscore QM’s future potential. Despite computational challenges, quantum mechanics (QM) and quantum computing are expected to be integral to drug discovery by 2030, driven by synergies between AI and QM, advancements in quantum technology, and industry adoption.

## Figures and Tables

**Table 1:** Comparison of quantum mechanical methods in drug discovery.

Method	Strengths	Limitations	Best Applications	Typical System Size	Computational Scaling	References
DFT	High accuracy for ground states; handles electron correlation; wide applicability	Expensive for large systems; functional dependence	Binding energies, electronic properties, and transition states	~500 atoms	O(N^3^)	[6,7]
HF	Fast convergence; reliable baseline; well-established theory	No electron correlation; poor for weak interactions	Initial geometries, charge distributions, and force field parameterization	~100 atoms	O(N^4^)	[2]
QM/MM	Combines QM accuracy with MM efficiency; handles large biomolecules	Complex boundary definitions; method-dependent accuracy	Enzyme catalysis, protein–ligand interactions	~10,000 atoms	O(N^3^) for QM region	[8]
FMO	Scalable to large systems; detailed interaction analysis	Fragmentation complexity approximates long-range effects	Protein–ligand binding decomposition, large biomolecules	Thousands of atoms	O(N^2^)	[9]

**Table 2:** Applications of quantum mechanics in drug discovery.

Application	Description	Key Methods	References
Molecular Modeling	Calculates electronic structures, geometries, and binding energies	DFT, Hartree–Fock, FMO	[11]
Biochemical Processes	Models enzyme catalysis and ligand interactions	QM/MM, DFT	[12]
Spectroscopic Analysis	Interprets NMR, IR, and UV-Vis data	DFT, semi-empirical	[13]
Force Field Development	Informs MD force fields	QM/MM, DFT	[4]
Property Prediction	Predicts ADMET reactivity	DFT, semi-empirical	[14]
Fragment-Based Drug Design	Evaluates fragments for lead optimization	DFT, FMO	[22]

**Table 3:** QM applications for major therapeutic areas.

Disease	Target Protein (PDB ID)	Current Drug Examples	Drug Limitations	QM Application Opportunities	Key QM Methods	References
COVID-19	SARS-CoV-2 Mpro (6LU7, 7BQY)	Nirmatrelvir (Paxlovid)	Off-target effects, resistance mutations	Covalent warhead optimization, resistance prediction	DFT, QM/MM	[26,27]
HIV	HIV-1 Protease (1HHP)	Ritonavir, Lopinavir	Drug resistance, side effects	Mutation-resistant inhibitor design	FMO, DFT	[15]
Cancer (Lung)	EGFR (2ITN, 4HJO)	Gefitinib, Erlotinib	Resistance mutations (T790M)	Mutant-selective inhibitor design	DFT, QM/MM	[25]
Cancer (Leukemia)	BCR-ABL (2HYY)	Imatinib, Nilotinib	Drug resistance, toxicity	Next-generation kinase inhibitors	DFT, FMO	[11,25]
Alzheimer’s Disease	BACE1 (4D8C)	None approved	Blood–brain barrier penetration	BBB-permeable inhibitor design	QM/MM, DFT	[28]
Malaria	Plasmodium Protease (1M43)	Artemisinin derivatives	Resistance emergence	Novel mechanism inhibitors	DFT, QM/MM	[29]
Tuberculosis	M. tuberculosis Protease (1Y5I)	Isoniazid, Rifampin	Multidrug resistance	Resistance-proof drug design	FMO, DFT	[29]

**Table 4:** Quantum computing platforms for drug discovery.

Platform	Company	Key Features	Drug Discovery Applications	Availability	References
IBM Quantum	IBM	127-qubit processors, Qiskit framework	VQE for small molecules, hybrid algorithms	Cloud access	[34]
Amazon Braket	Amazon	Multi-vendor access, D-Wave, IonQ, Rigetti	QAOA for virtual screening	Pay-per-use	[36]
Google Quantum AI	Google	Sycamore processor, Cirq framework	Molecular simulation research	Limited access	[31]
Azure Quantum	Microsoft	Quantum development kit, Q# language	Chemistry simulations	Developer preview	[36]

**Table 5:** Examples of QM-driven drug discovery.

Project	Therapeutic Area	QM Method	Outcome	References
SmartCADD	HIV	DFT, AI-QM, FMO	HIV drug candidates	[15]
SophosQM	Cancer	FMO, QM/MM	SMKI lead compounds	[11]
Pfizer-XtalPi CSP	General	DFT	Faster CSP	[17]
Imatinib/Nilotinib	Leukemia	QM docking	Market-ready drugs	[25]
PF-07321332	COVID-19	DFT	SARS-CoV-2 inhibitor	[26]

**Table 6:** QM-based drug discovery projects under development.

Project	Therapeutic Area	QM Method	Status	References
Aqemia	Cancer	QM-inspired algorithm	Preclinical trials by 2025	[33]
SmartCADD Expansion	Cancer, Neurodegenerative	DFT, AI-QM, FMO, quantum computing	Ongoing, October 2024	[15]
Algorithmiq Aurora	General	QM algorithms, quantum computing	Proof-of-concept, June 2023	[33]
Phytopharmaceuticals	Natural Products	QM holographic models	Early-stage, June 2025	[37]

**Table 7:** Binding affinity prediction accuracy comparison.

Method	Average Error (kcal/mol)	System Size Limit	Computational Time	Accuracy for Weak Interactions	References
Classical Docking	2.5–3.5	Unlimited	Minutes	Poor	[38]
MM/PBSA	1.5–2.5	~100,000 atoms	Hours	Moderate	[39]
DFT	0.5–1.5	~500 atoms	Hours–Days	Excellent	[11]
QM/MM	0.8–1.8	~10,000 atoms	Days	Very Good	[16]
FMO	1.0–2.0	Thousands of atoms	Days	Good	[11]

**Table 8:** Essential software tools for QM drug discovery.

Software Category	Tool Examples	License Type	Key Features	Cost (Academic/Commercial)	References
QM Packages	Gaussian 16, ORCA, Q-Chem	Commercial/Free	DFT, HF, post-HF methods	USD 1000–10,000/Free-5000	[18,20]
QM/MM	AMBER, CHARMM, NAMD	Mixed	Hybrid simulations	USD 500–2000/Free-1000	[4,8]
Visualization	VMD, PyMOL, ChimeraX	Free/Commercial	Molecular graphics	Free/USD 100–500	[13]
Quantum Computing	Qiskit, Cirq, PennyLane	Open-Source	Quantum algorithms	Free	[34]

## Data Availability

No new data were created or analyzed in this study.

## Supplementary Materials

The following supporting information can be downloaded at: https://www.mdpi.com/article/10.3390/ijms26136325/s1.

## Abbreviations

ADMET	Absorption, Distribution, Metabolism, Excretion, and Toxicity
AI	Artificial Intelligence
BSSE	Basis Set Superposition Error
CADD	Computer-Aided Drug Design
CDR	Complementarity-Determining Region
DFT	Density Functional Theory
FBDD	Fragment-Based Drug Discovery
FMO	Fragment Molecular Orbital
GPCR	G-Protein-Coupled Receptor
HF	Hartree–Fock
HPC	High-Performance Computing
IDP	Intrinsically Disordered Protein
LDA	Local Density Approximation
LNP	Lipid Nanoparticle
mAb	Monoclonal Antibody
MD	Molecular Dynamics
MM	Molecular Mechanics
MMP	Matrix Metalloproteinase
NISQ	Noisy Intermediate-Scale Quantum
NMR	Nuclear Magnetic Resonance
PDB	Protein Data Bank
QAOA	Quantum Approximate Optimization Algorithm
QM	Quantum Mechanics
QM/MM	Quantum Mechanics/Molecular Mechanics
QPE	Quantum Phase Estimation
SBDD	Structure-Based Drug Discovery
SCF	Self-Consistent Field
SMKI	Small-Molecule Kinase Inhibitor
VQE	Variational Quantum Eigensolver

## References

[1] Griffiths D.J., Schroeter D.F. (2018). Introduction to Quantum Mechanics.

[2] Szabo A., Ostlund N.S. (1996). Modern Quantum Chemistry: Introduction to Advanced Electronic Structure Theory.

[3] Born M., Oppenheimer J.R. (1927). Zur Quantentheorie der Moleküle. Ann. Phys..

[4] MacKerell A.D., Bashford D., Bellott M.L., Dunbrack R.L., Evanseck J.D., Field M.J., Fischer S., Gao J., Guo H., Ha S. (1998). All-atom empirical potential for molecular modeling and dynamics studies of proteins. J. Phys. Chem. B.

[5] Jensen F. (2017). Introduction to Computational Chemistry.

[6] Hohenberg P., Kohn W. (1964). Inhomogeneous electron gas. Phys. Rev..

[7] Parr R.G., Yang W. (1989). Density-Functional Theory of Atoms and Molecules.

[8] Senn H.M., Thiel W. (2009). QM/MM methods for biomolecular systems. Angew. Chem. Int. Ed..

[9] Kitaura K., Ikeo E., Asada T., Nakano T., Uebayasi M. (1999). Fragment molecular orbital method: An ab initio computational method for large biomolecular systems. Chem. Phys. Lett..

[10] Burke K. (2012). Perspective on density functional theory. J. Chem. Phys..

[11] Raha K., Peters M.B., Wang B., Yu N., Wollacott A.M., Westerhoff L.M., Merz K.M. (2007). The role of quantum mechanics in structure-based drug design. Drug Discov. Today.

[12] Kotev M., Sarrat L., Gonzalez C.D. (2020). User-friendly quantum mechanics: Applications for drug discovery. Methods Mol. Biol..

[13] Aucar M.G., Cavasotto C.N. (2020). Molecular docking using quantum mechanical-based methods. Methods Mol. Biol..

[14] van de Waterbeemd H., Gifford E. (2003). ADMET in silico modeling: Towards prediction paradise?. Nat. Rev. Drug Discov..

[15] Madushanka A., Laird E., Clark C., Kraka E. (2024). SmartCADD: AI-QM empowered drug discovery platform with explainability. J. Chem. Inf. Model..

[16] Misini Ignjatović M., Caldararu O., Dong G., Munoz-Gutierrez C., Adasme-Carreno F., Ryde U. (2022). Binding-affinity predictions of HSP90 in the D3R grand challenge 2015 with docking, MM/GBSA, QM/MM, and free-energy simulations. J. Comput.-Aided Mol. Des..

[17] Hancock B. (2018). How Quantum Physics and AI Are Disrupting Drug Discovery and Development.

[18] Frisch M.J. (2016). Gaussian 16 User’s Reference Manual.

[19] Warshel A., Levitt M. (1976). Theoretical studies of enzymic reactions: Dielectric, electrostatic and steric stabilization of the carbonium ion in the reaction of lysozyme. J. Mol. Biol..

[20] Leach A.R. (2001). Molecular Modelling: Principles and Applications.

[21] Perakakis P. (2020). Open science in drug discovery: Addressing the challenges of data sharing. Drug Discov. Today.

[22] Murray C.W., Rees D.C. (2009). The rise of fragment-based drug discovery. Nat. Chem..

[23] Arodola O.A., Soliman M.E. (2017). Quantum mechanics implementation in drug-design workflows: Does it really help?. Drug Des. Dev. Ther..

[24] Fedorov D.G., Kitaura K. (2009). The Fragment Molecular Orbital Method: Practical Applications to Large Molecular Systems.

[25] Kim S., Lee Y., Song B.R., Sim H., Kang E.H., Hwang M., Yu N., Hong S., Park C., Ahn B.-C. (2024). Drug Response of Patient-Derived Lung Cancer Cells Predicts Clinical Outcomes of Targeted Therapy. Cancers.

[26] Owen D.R., Allerton C.M.N., Anderson A.S., Aschenbrenner L., Avery M., Berritt S., Boras B., Cardin R.D., Carlo A., Coffman K.J. (2021). An oral SARS-CoV-2 Mpro inhibitor clinical candidate for the treatment of COVID-19. Science.

[27] Schoenfeld D. (2021). Lipid nanoparticles for mRNA delivery. Nat. Rev. Drug Discov..

[28] Tambuyzer E., Vandendriessche B., Austin C.P., Brooks P.J., Larsson K., Needleman K.I.M., Valentine J., Davies K., Groft S.C., Preti R. (2020). Therapies for rare diseases: Therapeutic modalities, progress and challenges ahead. Nat. Rev. Drug Discov..

[29] Pushpakom S., Iorio F., Eyers P.A., Escott K.J., Hopper S., Wells A., Doig A., Guilliams T., Latimer J., McNamee C. (2019). Drug repurposing: Progress, challenges and recommendations. Nat. Rev. Drug Discov..

[30] Nielsen M.A., Chuang I.L. (2010). Quantum Computation and Quantum Information.

[31] Kim Y. (2023). Evidence for quantum advantage in chemical simulation with a near-term quantum device. Nature.

[32] Srivastava A. (2023). Quantum computing in drug discovery. Inf. Syst. Smart City.

[33] Osorio A.V. (2024). 14 Companies Using Quantum Theory to Accelerate Drug Discovery.

[34] McArdle S., Endo S., Aspuru-Guzik A., Benjamin S.C., Yuan X. (2020). Quantum computational chemistry. Rev. Mod. Phys..

[35] Jorgensen W.L. (2004). The many roles of computation in drug discovery. Science.

[36] Biamonte J., Wittek P., Pancotti N., Rebentrost P., Wiebe N., Lloyd S. (2017). Quantum machine learning. Nature.

[37] Naik S.Y., Darshan B.R., Patil C., Goudanavar P. (2025). Quantum-inspired computational drug design for phytopharmaceuticals: A herbal holography analysis. J. Mol. Model..

[38] Shoichet B.K. (2004). Virtual screening of chemical libraries. Nature.

[39] Friesner R.A., Banks J.L., Murphy R.B., Halgren T.A., Klicic J.J., Mainz D.T., Repasky M.P., Knoll E.H., Shelley M., Perry J.K. (2004). Glide: A new approach for rapid, accurate docking and scoring. J. Med. Chem..

[40] Isert C., Atz K., Jiménez-Luna J., Schneider G. (2022). QMugs, quantum mechanical properties of drug-like molecules. Sci. Data.

[41] Cavasotto C.N., Sadybekov A.V., Katritch V. (2023). Computational approaches streamlining drug discovery. Nature.

[42] Behler J. (2011). Neural network potential-energy surfaces in chemistry: A tool for large-scale simulations. Phys. Chem. Chem. Phys..

[43] Wang D., Tai P.W.L., Gao G. (2020). Adeno-associated virus vectors for gene therapy. Mol. Ther..

[44] McGhee P. (2018). Analytical challenges in biosimilar development. BioDrugs.

[45] Barducci A. (2011). Metadynamics: A method for exploring free-energy landscapes. Wiley Interdiscip. Rev. Comput. Mol. Sci..

[46] Cramer R.D. (2019). Computational chemistry education: A global perspective. J. Chem. Inf. Model..

[47] Miller B.R. (2020). The Molecular Sciences Software Institute: Building a community for computational molecular science. J. Chem. Inf. Model..

