# Supplementary material for: Quantum Mechanics in Drug Discovery: A Comprehensive Review of Methods, Applications, and Future Directions
Source: Int J Mol Sci. 2025 Jun 30;26(13):6325. doi: 10.3390/ijms26136325 (PMC12249871; doi:10.3390/ijms26136325)
Supplement: Supplementary file 1 [file ijms-26-06325-s001.zip › ijms-3730353-supplementary.pdf]

Equation S1: Time-Independent Schrödinger Equation

Mathematical Expression:

$\hat{H}\psi = E\psi$

Description:

The fundamental equation of quantum mechanics that describes the wave function of a quantum system. This equation forms the foundation for all quantum mechanical calculations in drug discovery.

Terms and Symbols:

- **$\hat{H}$** = Hamiltonian operator (total energy operator)
- **ψ** = Wave function (probability amplitude distribution)
- **E** = Energy eigenvalue

Application in Drug Discovery:

This equation is the starting point for all quantum mechanical calculations of molecular systems, including drug-target interactions, binding affinity predictions, and electronic structure analysis.

Equation S2: Hamiltonian Operator

Mathematical Expression:

$\hat{H} = -\hbar^2/2m \nabla^2 + V(x)$

Description:

The Hamiltonian operator represents the total energy of a quantum system, combining kinetic and potential energy terms. Essential for understanding molecular interactions in drug discovery.

Terms and Symbols:

- **$\hbar$** = Reduced Planck constant (h/2π)
- **m** = Particle mass

- **∇^2** = Laplacian operator (second derivative)
- **V(x)** = Potential energy function

Application in Drug Discovery:

Used to calculate molecular energies, optimize drug geometries, and predict reaction pathways in enzyme-catalyzed processes.

Equation S3: Born-Oppenheimer Approximation

Mathematical Expression:

$\hat{H}_e \psi_e(r;R) = E_e(R)\psi_e(r;R)$

Description:

Separates electronic and nuclear motion by treating nuclei as stationary. This approximation is crucial for making quantum mechanical calculations tractable in molecular systems.

Terms and Symbols:

- **$\hat{H}_e$** = Electronic Hamiltonian
- **ψ_e** = Electronic wave function
- **r** = Electron coordinates
- **R** = Nuclear coordinates
- **E_e(R)** = Electronic energy as function of nuclear positions

Application in Drug Discovery:

Enables the calculation of potential energy surfaces for drug-receptor interactions and allows separation of electronic structure calculations from nuclear dynamics.

Equation S4: DFT Energy Functional

Mathematical Expression:

$E[\rho] = T[\rho] + V_{ext}[\rho] + V_{ee}[\rho] + E_xc[\rho]$

Description:

Density Functional Theory (DFT) expresses the total energy as a functional of electron density. This is the most widely used method in computational drug discovery due to its balance of accuracy and efficiency.

Terms and Symbols:

- **$E[\rho]$** = Total energy functional
- **$T[\rho]$** = Kinetic energy of non-interacting electrons
- **$V_{ext}[\rho]$** = External potential energy
- **$V_{ee}[\rho]$** = Classical electron-electron repulsion
- **$E_{x}c[\rho]$** = Exchange-correlation energy

Application in Drug Discovery:

Most commonly used method for calculating binding energies, molecular properties, and electronic structures in drug design. Used in platforms like SmartCADD and SophosQM.

Equation S5: Kohn-Sham Equations

Mathematical Expression:

$$[-\hbar^2/2m\ \nabla^2 + v_{e}ff(r)]\varphi_i(r) = \varepsilon_i\varphi_i(r)$$

Description:

The Kohn-Sham equations transform the many-electron problem into a set of single-electron equations. These are solved self-consistently to obtain the electron density and total energy in DFT calculations.

Terms and Symbols:

- **$\varphi_i(r)$** = Kohn-Sham orbitals
- **ε_i** = Kohn-Sham orbital energies
- **$v_{e}ff(r)$** = Effective potential (includes external, Hartree, and exchange-correlation)

Application in Drug Discovery:

Core computational engine of DFT calculations, used to determine molecular orbitals, electron densities, and electronic properties critical for drug design.

Equation S6: Hartree-Fock Energy Expression

Mathematical Expression:

$E\_HF = \langle \Psi\_HF | \hat{H} | \Psi\_HF \rangle$

Description:

The Hartree-Fock energy is the expectation value of the Hamiltonian with respect to the Hartree-Fock wave function. This provides a mean-field approximation to the many-electron problem.

Terms and Symbols:

- **E_HF** = Hartree-Fock energy
- **Ψ_HF** = Hartree-Fock wave function (single Slater determinant)
- **⟨ | ⟩** = Expectation value (quantum mechanical average)

Application in Drug Discovery:

Provides baseline electronic structures for small molecules, often used as starting point for more accurate methods or for initial structure optimization in drug design.

Equation S7: Hartree-Fock Equations

Mathematical Expression:

$\hat{f}\varphi_i = \varepsilon_i\varphi_i$

Description:

The Hartree-Fock equations are eigenvalue equations for molecular orbitals. The Fock operator includes kinetic energy, nuclear attraction, and electron-electron repulsion terms.

Terms and Symbols:

- **$\hat{f}$** = Fock operator (effective one-electron Hamiltonian)
- **φ_i** = Molecular orbitals

- **ε_i** = Orbital energies

Application in Drug Discovery:

Used to calculate molecular orbitals and electronic properties, particularly useful for understanding charge distributions and electronic interactions in drug-receptor complexes.

Equation S8: QM/MM Total Energy

Mathematical Expression:

$E_{total} = E_{QM} + E_{MM} + E_{QM/MM}$

Description:

The QM/MM method combines quantum mechanical treatment of the active region with classical molecular mechanics for the environment. This allows accurate modeling of large biomolecular systems.

Terms and Symbols:

- **E_{QM}** = Energy of quantum mechanical region
- **E_{MM}** = Energy of molecular mechanics region
- **$E_{QM/MM}$** = Interaction energy between QM and MM regions

Application in Drug Discovery:

Essential for studying enzyme-catalyzed reactions, protein-ligand binding, and large biomolecular systems where full QM treatment is computationally prohibitive.

Equation S9: QM/MM Hamiltonian

Mathematical Expression:

$\hat{H}_{QM/MM} = \hat{H}_{QM} + \Sigma_i \Sigma_k q_k/|r_i - R_k|$

Description:

The QM/MM Hamiltonian includes the interaction between QM electrons and MM point charges. This embedding allows the QM region to respond to the electrostatic environment of the MM region.

Terms and Symbols:

- **$\hat{H}_{QM}$** = Quantum mechanical Hamiltonian
- **q_k** = Point charges in MM region
- **r_i** = QM electron coordinates
- **R_k** = MM atom positions

Application in Drug Discovery:

Enables accurate modeling of drug-protein interactions where the drug and binding site are treated quantum mechanically while the rest of the protein uses classical mechanics.

Equation S10: Fragment Molecular Orbital (FMO) Energy

Mathematical Expression:

$E_{FMO} = \Sigma_i E'_i + \Sigma_i \Sigma_{j>i} \Delta E'_{ij}$

Description:

The FMO method divides large molecules into fragments and calculates their interactions. This allows quantum mechanical analysis of very large biomolecular systems while maintaining reasonable computational cost.

Terms and Symbols:

- **E'_i** = Energy of fragment i in the field of other fragments
- **$\Delta E'_{ij}$** = Interaction energy between fragments i and j
- **Σ_i** = Sum over all fragments
- **$\Sigma_{j>i}$** = Sum over all unique fragment pairs

Application in Drug Discovery:

Particularly useful for analyzing protein-ligand binding interactions, decomposing binding energies into residue-specific contributions, and studying large biomolecular complexes.

Equation S11: FMO Fragment Hamiltonian

Mathematical Expression:

$\hat{H}'_i = \hat{H}_i + V^{ESP}_i$

Description:

Each fragment in FMO is solved in the electrostatic field of all other fragments. This creates a self-consistent treatment where fragments mutually polarize each other.

Terms and Symbols:

- **$\hat{H}'_i$** = Fragment Hamiltonian including environmental effects
- **$\hat{H}_i$** = Isolated fragment Hamiltonian
- **V^{ESP}_i** = Electrostatic potential from all other fragments

Application in Drug Discovery:

Enables detailed analysis of how different protein residues contribute to drug binding, helping identify key interactions for lead optimization and rational drug design.

Summary and Applications

These eleven fundamental equations form the mathematical foundation of quantum mechanics in drug discovery:

1. **Small Molecule Design**: Equations 1-7 are primarily used for small molecule optimization and property prediction.

2. **Large Biomolecular Systems**: Equations 8-11 enable the study of drug-protein interactions and enzyme mechanisms.

3. **Computational Efficiency**: The progression from full QM (Equations 1-7) to hybrid methods (QM/MM, FMO) reflects the balance between accuracy and computational feasibility.

4. **Industry Applications**: These methods are implemented in platforms like SmartCADD, SophosQM, and Gaussian, driving modern drug discovery pipelines.

5. **Future Developments**: Integration with quantum computing and AI is expected to enhance the speed and accuracy of these calculations by 2030-2035.

This comprehensive set of equations provides the theoretical framework for understanding and predicting molecular behavior in drug discovery, from initial lead identification to final optimization of therapeutic compounds.
